# Identification of a novel gene signature with regard to ferroptosis, prognosis prediction, and immune microenvironment in osteosarcoma

**DOI:** 10.3389/fgene.2022.944978

**Published:** 2022-10-18

**Authors:** Di Zheng, Kezhou Xia, Zhun Wei, Zicheng Wei, Weichun Guo

**Affiliations:** Department of Orthopedics, Renmin Hospital of Wuhan University, Wuhan, China

**Keywords:** ferroptosis, RSL3, osteosarcoma, prognosis, immune microenvironment

## Abstract

Ferroptosis is a novel form of non-apoptotic cell death that mainly results from the iron-dependent lethal accumulation of lipid peroxidation products. Here, we defined differentially expressed genes between control and RSL3-treated osteosarcoma cells as ferroptosis-associated genes (FAGs). These FAGs were then subjected to weighted gene correlation network analysis (WGCNA), and we found that the turquoise module, containing 71 FAGs, was markedly related to the patient’s vital status. After that, FAGs in the turquoise module were utilized to construct a prognostic multigene (*COL5A2*, *HOXB4*, and *UNC5B*) signature for risk stratification in osteosarcoma. Validation in internal and external cohorts indicated the accuracy and clinical applicability of this signature in predicting the prognosis of patients with osteosarcoma. Univariate and multivariate Cox regression analyses suggested that the signature-derived risk score is an independent indicator of patient prognosis. Immunological analysis indicated that significant variations in stromal and ESTIMATE scores, as well as tumor purity, were found when the high- and low-risk groups were compared. Regarding immune cell infiltration, the proportion of activated CD4 memory T cells was significantly lower in the high-risk group than that in the low-risk group. The ssGSEA results suggested that CD8^+^ T, Tfh, and Th1 cell scores were consistently lower in the high-risk group than those in the low-risk group. In terms of immune-related activities, the high-risk group had considerably lower scores for promoting inflammation, T-cell co-inhibition, and T-cell co-stimulation than the low-risk group, indicating the differential immunological state of the high- and low-risk groups. Of the three FAGs included in the signature, the expression of *COL5A2*, *HOXB4*, and *UNC5B* was higher in the high-risk groups, and the expression of *COL5A2* and *UNC5B* was negatively associated with patient prognosis. Additionally, the mRNA levels of *COL5A2* and *HOXB4* were lower and those of *UNC5B* were higher in RSL3-treated cells than in control cells. In all, we systematically analyzed the transcriptional changes of osteosarcoma cells induced by RSL3 and constructed a novel three-gene signature with regard to ferroptosis, prognosis prediction, and immune microenvironment. We also identified *COL5A2*, *HOXB4*, and *UNC5B* as potential therapeutic targets and important regulators of ferroptosis in osteosarcoma.

## 1 Introduction

Osteosarcoma (OS) is the most common type of malignant bone tumor that primarily affects the metaphysis of weight-bearing long bones such as the proximal humerus, distal femur, and proximal tibia (Ritter and Bielack, 2010). It is a rare illness with an annual incidence of approximately 1–3 cases per million, accounting for approximately 20% of all primary malignant bone tumors (Kansara et al., 2014; Hernandez Tejada et al., 2020). Osteosarcoma has a characteristic age distribution, with peak incidences in young adolescents and those over the age of 50 (Cole et al., 2022; Santos et al., 2022). The conventional treatment plan for osteosarcoma is the surgical removal of visible tumors combined with neoadjuvant chemotherapy, which has increased the 5-year survival rate of patients with localized malignancies to 60–70% (Whelan and Davis, 2018; Gill and Gorlick, 2021). However, for those with recurrent or metastatic osteosarcoma, the 5-year survival rate dramatically decreases to less than 20% (Sheng et al., 2021). Furthermore, because of the substantial genetic variability of osteosarcoma (Rickel et al., 2017; Belayneh et al., 2021), prognosis differs even when patients have identical clinical characteristics and are receiving standard therapy. The development of innovative therapeutic techniques and the identification of reliable prognostic indicators are critical for improving patient clinical outcomes.

Ferroptosis, first defined by Dixon in 2012, is a unique form of iron-dependent non-apoptotic cell death characterized by the lethal accumulation of lipid peroxidation (Dixon et al., 2012; Li et al., 2020a). Ferroptosis has unique pathophysiological characteristics and biological processes that differ from other forms of programmed cell death, including apoptosis, necrosis, and autophagy (Mou et al., 2019; Tang et al., 2020a). Morphologically, cells undergoing ferroptosis exhibit shrinkage of mitochondria, with a reduced number of mitochondrial cristae and increased mitochondrial density (Tang et al., 2020b). Biochemically, lipid peroxides cannot be reduced, owing to the depletion of intracellular GSH and inactivation of glutathione peroxidases (GPXs). In contrast, iron accumulation promotes lipid oxidation in a Fenton-like manner, resulting in the accumulation of cytotoxic lipid peroxidation products, further inducing ferroptosis (Conrad and Pratt, 2019). Genes regulating iron homeostasis, energy metabolism, and lipid peroxidation metabolism are closely associated with ferroptosis (Hassannia et al., 2019). Previous studies have shown that ferroptosis plays a vital role in the occurrence and development of diseases including tumors (Conrad et al., 2020; Fu et al., 2020). Ferroptosis is regarded as a novel method for killing therapy-resistant tumors (Song et al., 2020). *In vitro* experiments have suggested that osteosarcoma cells are sensitive to ferroptosis inducers, and some molecules could increase chemosensitivity, at least partly, by inducing ferroptosis (Dixon et al., 2012; Lv et al., 2020; Lin et al., 2021). However, the regulatory mechanism of ferroptosis in osteosarcoma and its clinical significance remain unclear.

In the present study, we defined differentially expressed genes between control and RSL3-treated osteosarcoma cells as ferroptosis-associated genes (FAGs) in osteosarcoma. The expression profiles of these FAGs were retrieved from the dataset of The Cancer Genome Atlas (TCGA) osteosarcoma cohort. Next, module genes that were significantly correlated with vital status were identified through weighted gene correlation network analysis (WGCNA) and utilized to construct a prognostic multigene signature in osteosarcoma. Subsequently, the accuracy and specificity of the signature were validated in both internal and external cohorts. Additionally, we explored the association of this signature with the osteosarcoma immune microenvironment and immune cell infiltration. Finally, we performed the Kaplan–Meier survival analysis of the FAGs in the signature and compared their transcript levels in the control and RSL3-treated cells and different risk groups.

## 2 Materials and methods

### 2.1 Data acquisition and procession

We downloaded osteosarcoma-related RNA-seq (FPKM) expression profiles and corresponding clinical annotations from The Cancer Genome Atlas (TCGA) database. The GSE21257 dataset comprising the RNA array data on 53 osteosarcoma samples and clinical information was obtained from the Gene Expression Omnibus (GEO) database and used for external validation. We deleted cases with incomplete clinical information and ultimately obtained 139 cases, including 86 from TCGA database and 53 from the GEO database for subsequent analysis.

### 2.2 Cell culture and death assays

Osteosarcoma cell lines (143B and U2OS) were obtained from the American Type Culture Collection (ATCC, Manassas, VA). The 143B and U2OS cells were cultured in α-MEM or McCoy’s 5a medium (HyClone, United States), respectively. All the media were supplemented with 10% fetal bovine serum (Gibco, United States), 100 U/mL penicillin, and 100 mg/ml streptomycin (Invitrogen, Grand Island, NY, United States). All the human osteosarcoma cell lines were maintained in a humidified incubator at 37°C with a 5% CO_2_ atmosphere. Cell death after RSL3 treatment was assessed *via* propidium iodide (PI) staining. Briefly, cells treated with DMSO or RSL3 (1 µM) for 24 h were washed three times with ice-cold PBS and were then stained with 2.5 μg/ml PI (Sigma-Aldrich, St. Louis, MO, United States) for 30 min at room temperature. The stained cells were observed with a fluorescence microscope (Olympus, Tokyo, Japan).

### 2.3 Transmission electron microscopy

The 143B cells were seeded onto 6-cm plates and were treated with RSL3 (1 uM) for 24 h. Images were acquired using a transmission electron microscope (Hitachi; HT7700, Japan); all fields were evaluated at ×12.0 k magnification.

### 2.4 RNA isolation, RNA-sequence, and qRT-PCR

The 143B and U2OS cells were treated with RSL3 (1 μM) for 24 h to induce ferroptosis before being collected for RNA isolation. TRIzol reagent (Invitrogen, Carlsbad, CA, United States) was used to extract the total RNA from the control and RSL3-treated 143B cells, according to the manufacturer’s instructions. The extracted RNA was quantified and qualified using the Agilent 2100 system (Agilent Technologies, Santa Clara, CA, United States). A total of 5 μg RNA in each sample (*n* = 3/group) was transferred to BGI (BGI Group, Shenzhen, China) for RNA sequencing. Thereafter, library preparation was initiated. Subsequently, 2 × 50 bp pair-end RNA sequencing was performed on the BGISEQ-500 platform according to the manufacturer’s protocol. The quality of the sequencing reads was evaluated and subsequently aligned to the human reference genome build 37 (hg19), as previously described (Logie et al., 2021). For subsequent investigation, RNA-seq data in the FPKM format were used. For quantitative real-time polymerase chain reaction (qRT-PCR), 1 μg total RNA was used to synthesize cDNA using the RevertAid First Strand cDNA Synthesis Kit (Thermo Fisher Scientific, Waltham, MA), and SYBR Green Mix (Vazyme, Nanjing, China) was used to detect the relative expression levels of target genes. The primer sequences were as follows: *GAPDH*, forward 5′-GGA​AGC​TTG​TCA​TCA​ATG​GAA​ATC-3′ and reverse 5′-TGA​TGA​CCC​TTT​TGG​CTC​CC-3′; *COL5A2*, forward 5′-CAG​GGT​TTA​CAA​GGA​CAG​CAA​G-3′ and reverse 5′-AGG​GCC​TTC​AAG​ACC​TTT​GTG-3′; *HOXB4*, forward 5′-TTCGTGCCCATTCACTGAGG-3′ and reverse 5′-CCG​GGT​CTC​TGA​GTC​TCT​CT-3′; and *UNC5B*, forward 5′-CTG​GCA​CAT​ACC​CTA​GCG​ATT-3′ and reverse 5′-CTC​AAT​ACT​GTC​TGG​GTC​CCT​TCT-3′. All the experiments were repeated with at least three independent biological replicates.

### 2.5 Identification of ferroptosis-associated genes in osteosarcoma and functional annotation

The *edgeR* package (Robinson et al., 2010) in R software was used to identify differentially expressed genes (DEGs) between the control and RSL3-treated cells, with the selection criteria of *p* < 0.05 and | log_2_FC | >1, and these DEGs were recognized as ferroptosis-associated genes (FAGs) in osteosarcoma. These FAGs were subjected to Gene Ontology (GO) and Kyoto Encyclopedia of Genes and Genomes (KEGG) pathway enrichment analyses using the *clusterProfiler* package (Yu et al., 2012).

### 2.6 Weighted gene correlation network analysis

The expression profiles of the FAGs in TCGA cohort were obtained and used to construct a weighted gene correlation network, as previously described. Briefly, hierarchical clustering analysis of the expression profile of osteosarcoma samples with multiple clinical features (age, sex, OS, and censor) was performed to exclude outliers. The best soft threshold power (β) was then filtered out using the *WGCNA* package (Langfelder and Horvath, 2008) to ensure the construction of scale-free networks. The adjacency matrix of the FAGs’ expression data was calculated based on the soft threshold power and correlation coefficients of these FAGs and was then transformed into a topological overlap matrix (TOM). Subsequently, average linkage hierarchical clustering was conducted to classify the FAGs with similar expression modes into the same modules according to the TOM-based dissimilarity measure, with the mini-size of module gene numbers set as 10. Finally, Pearson’s correlation analysis was performed to establish the relationship between the modules and clinical traits. Modules with *p* < 0.05 were regarded as significantly associated with the vital status. In this study, the FAGs in the turquoise module were selected for subsequent research (Figure 2E).

### 2.7 Construction and validation of a FAG-based signature in osteosarcoma

To create a prognostic signature for osteosarcoma, TCGA cohort (entire cohort) was randomly divided into training and testing cohorts in a 1:1 ratio. In the training cohort, FAGs in the turquoise module were subjected to least absolute shrinkage and selection operator (LASSO) regression analysis using the *glmnet* package (Engebretsen and Bohlin, 2019) in R to exclude overlapping genes. Subsequently, multivariate Cox regression analysis was used to screen for independent prognostic genes from the LASSO analysis’ remaining robust genes and generate regression coefficients for the corresponding genes. The signature was built using a linear combination of the gene expression level and regression coefficient, and the formula for calculating the patients’ risk score based on the signature was as follows: Risk score = $\overset{n}{\underset{i=1}{\sum }}\left(Coefi*Expi\right)$ . The Coefi value is the regression coefficient of the selected FAGs, and the Expi value represents the gene expression level of the selected FAGs. The risk score of each patient in the training, testing, entire, and GSE21257 cohorts was then calculated using the “predict” function in R software, which allowed patients to be classified as high- or low-risk groups based on the median risk score value in the training cohort. Kaplan–Meier survival curves were used to assess overall survival (OS) across different risk categories in the training, testing, whole, and GSE21257 cohorts. Time-dependent receiver operating characteristic (ROC) analysis was performed to assess the accuracy and specificity of the signature for prognosis prediction.

### 2.8 Construction and assessment of the nomogram

We used the *rms* package (Zhang et al., 2019) in R software to build a prognostic nomogram that integrated the signature-derived risk score and multiple clinical characteristics including sex and age. Calibration curves were drawn to evaluate the consistency of nomogram-predicted OS and the actual survival probability of patients with osteosarcoma.

### 2.9 Gene set enrichment analysis

GSEA4.0.2 software was used for gene set enrichment analysis, which was obtained from an online website (http://www.gsea-msigdb.org/gsea/downloads). In several studies, gene expression matrices have been separated into various groups, including control or RSL3-treated groups and high- or low-risk groups. Pathways were considered considerably enriched if they met the *p-*value < 0.05 and |NES| >1 selection criteria.

### 2.10 Immune landscape difference between high- and low-risk groups

The ESTIMATE (Estimation of STromal and Immune cells in MAlignant Tumor tissues using Expression data) algorithm (through the *estimate* package in R) was utilized to quantify immune, stromal, and estimate scores, as well as tumor purity, using gene expression matrices (Xu et al., 2021). The CIBERSORT algorithm was employed to calculate the proportion of 22 infiltrating immune cell types in each osteosarcoma sample (Newman et al., 2015). Single-sample gene set enrichment analysis (ssGSEA) was performed to explore the scores of immune cells and immune-related functions using the *GSVA* package in R (Xiao et al., 2020). All the aforementioned results were then compared between the high- and low-risk groups to explore the immune landscape differences between the two risk groups.

### 2.11 Statistical analysis

All statistical analyses were conducted by R 4.1.0 and GraphPad Prism 8. Student’s t-test and Wilcoxon test were used to compare the differences in the variables between the two risk groups or the control and RSL3-treated groups. The Kaplan–Meier method and log-rank test were used to evaluate the difference in OS between the high- and low-risk groups. Statistical significance was set at *p* < 0.05.

## 3 Results

### 3.1 Transcriptional changes induced by RSL3 and functional analyses

**FIGURE 1:**
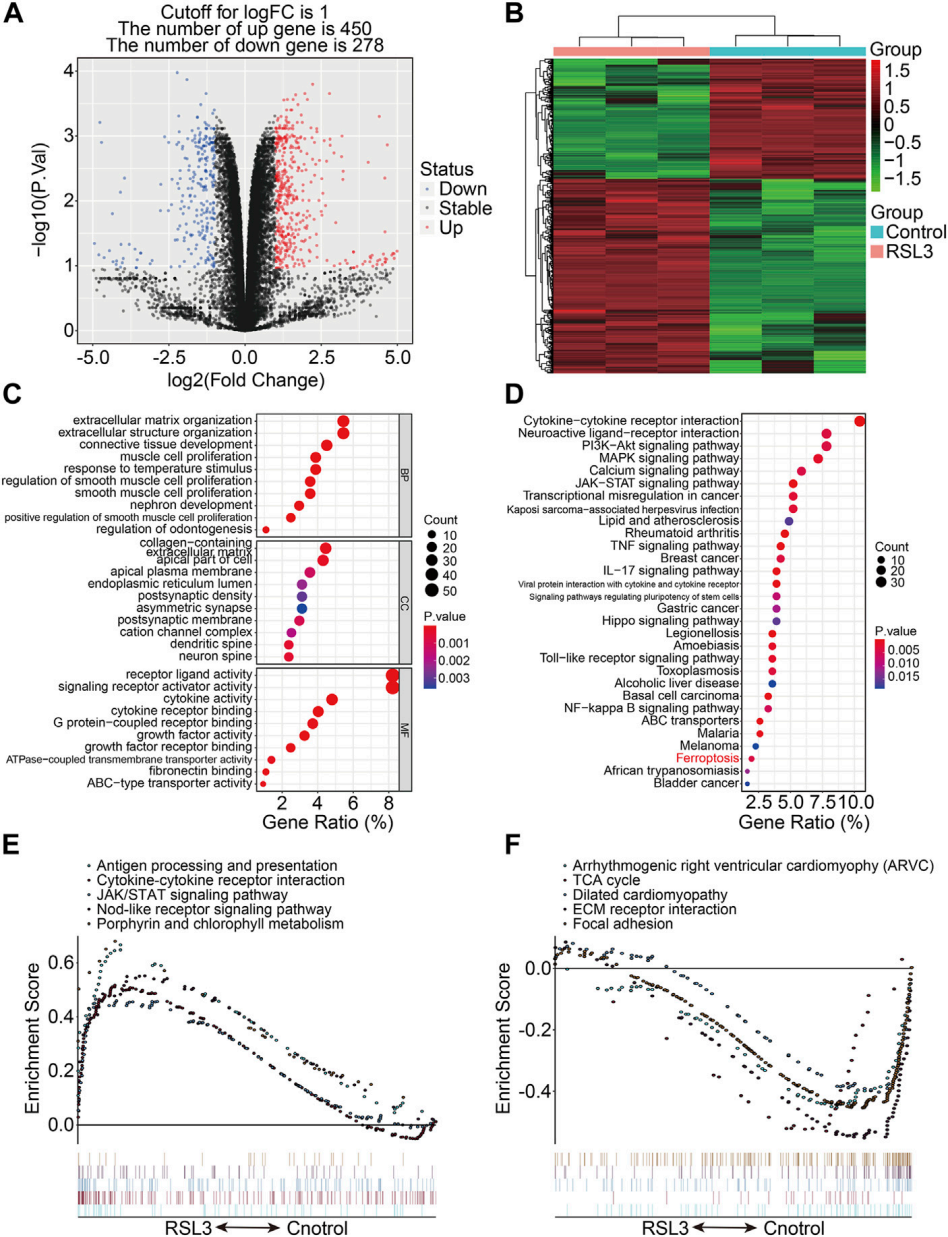
Transcriptional changes induced by RSL3 and functional analyses. **(A)** Volcano plot represents genes differentially expressed between RLS3-treated cells and control cells. **(B)** Heatmap for the expression profile of ferroptosis-associated genes (FAGs). **(C,D)** GO and KEGG analyses of FAGs. **(E,F)** Gene set enrichment analysis.

RSL3, an inhibitor of glutathione peroxidase 4 (GPX4), was used to induce ferroptosis in 143B cells, as was confirmed by the increased number of PI-positive cells and morphological features (Supplementary Figure S1). To describe the transcriptional alterations in ferroptotic 143B cells, we performed RNA sequencing analysis of RSL3-treated and control cells. With the selection criteria of p-value < 0.05 and | log_2_FC | >1, a total of 728 genes that were differentially expressed between the RLS3-treated cells and control cells were screened out, and these genes were regarded as ferroptosis-associated genes (FAGs) in osteosarcoma. Of the 728 FAGs, 450 and 278 genes were upregulated and downregulated, respectively, in the RSL3-treated cells (Figure 1A). Figure 1B shows the expression profiles of these FAGs in the RSL3-treated and control cells. Subsequently, we conducted GO and KEGG functional enrichment analyses of 728 FAGs. In terms of biological processes, FAGs were primarily enriched in extracellular matrix organization, extracellular structure organization, and connective tissue development. As for the cellular component, the collagen-containing extracellular matrix, apical part of the cell, and apical plasma membrane were the three most enriched terms. In the molecular function category, FAGs were mainly enriched in receptor ligand, signaling receptor activator, and cytokine activities (Figure 1C). The KEGG enrichment analysis indicated that PI3K-Akt, MAPK, calcium, JAK-STAT, TNF, IL-17, Hippo, and NF-kappa B signaling pathways and the ferroptosis pathway were significantly enriched (Figure 1D). The gene set enrichment analysis revealed that pathways including antigen processing and presentation, the cytokine–cytokine receptor interaction, JAK/STAT signaling pathway, nod-like receptor signaling pathway, and porphyrin and chlorophyll metabolism were significantly enriched in the RSL3-treated group (Figure 1E), whereas pathways including the TCA cycle, ECM receptor interaction, and focal adhesion were negatively associated with treatment with the ferroptosis inducer RSL3 (Figure 1F).

### 3.2 WGCNA identified modules related to the prognosis of osteosarcoma

**FIGURE 2:**
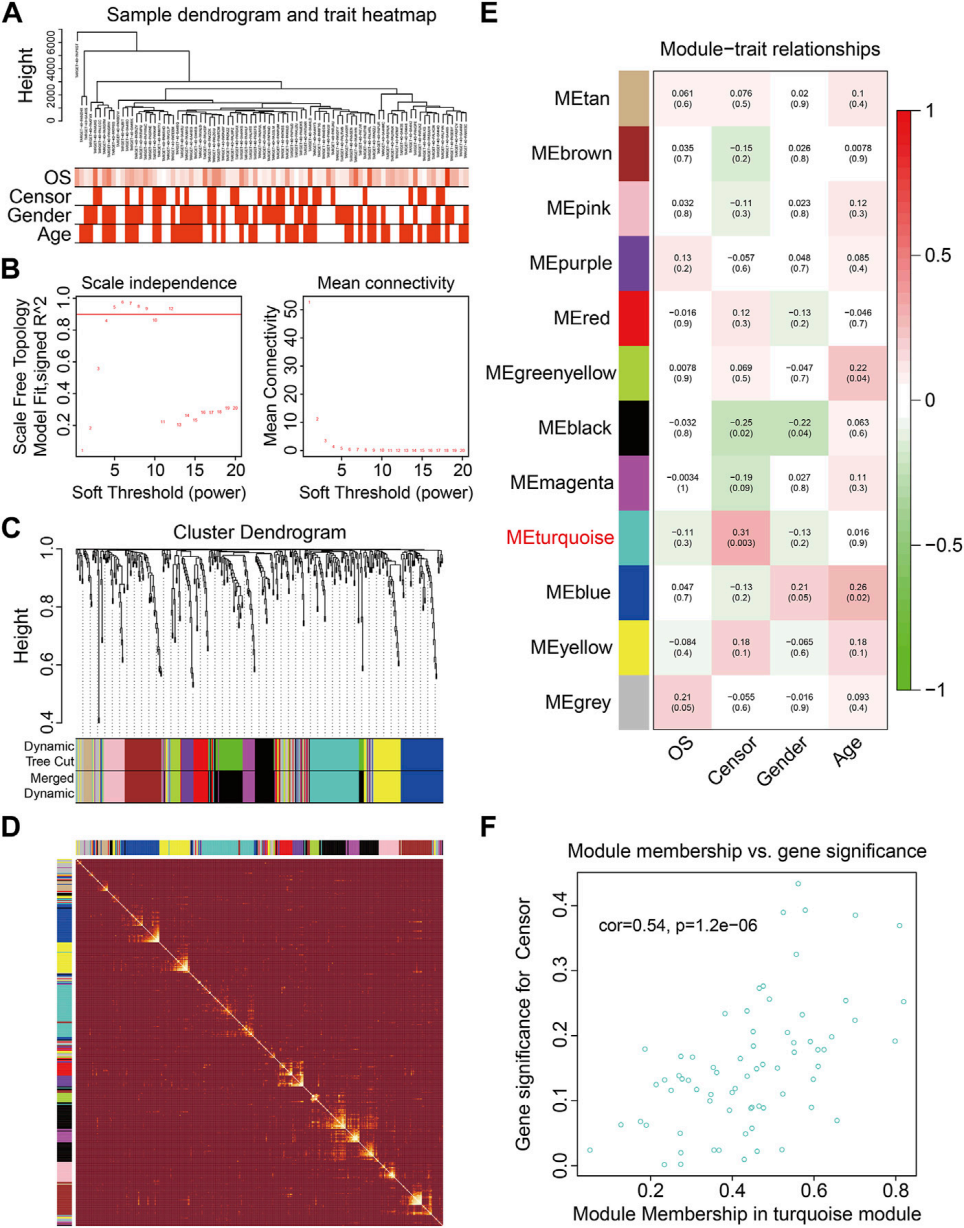
Weighted gene coexpression network analysis of the differentially expressed genes induced by RSL3. **(A)** Cluster analysis between the FAGs in TCGA osteosarcoma dataset and clinical traits. **(B)** Screening and validation of the soft threshold. **(C)** Cluster dendrogram indicating diverse FAG modules. **(D)** Correlation heatmap of genes in different modules. **(E)** Correlation analysis between gene modules and clinical features such as gender, age, censor, and overall survival (OS) time. **(F)** Scatter plot shows the module membership between the turquoise module and clinical feature vital status.

To identify genes related to the prognosis of osteosarcoma, the WGCNA algorithm was employed to construct a co-expression network using the expression data profile of these FAGs from TCGA osteosarcoma dataset. First, 86 osteosarcoma samples with different genders, ages, and OS rates were selected for FAG expression clustering (Figure 2A). We selected β = 4 as the soft thresholding parameter to build the scale-free network (Figure 2B). Using a merged dynamic tree cut, we identified 12 gene co-expression modules by setting the minimum cluster size to 10 (Figure 2C). A correlation heatmap was constructed to analyze the relationships between the 12 modules, and it was suggested that genes within the module were highly correlated (Figure 2D). Subsequently, we analyzed the associations between the gene modules and clinical features such as sex, age, vital status (censor), and OS time. As shown in Figure 2E, the green–yellow (R = 0.22 and *p* = 0.04) and blue modules (R = 0.26 and *p* = 0.02) were significantly correlated with the age of the patients with osteosarcoma. The turquoise module containing 71 FAGs was markedly associated with the vital status of patients with osteosarcoma (R = 0.31 and *p* = 0.003). The scatter plot also suggested that the module membership of the turquoise module was positively correlated with gene significance for the vital status (Figure 2F), further indicating the relationship between the turquoise module and the prognosis of osteosarcoma patients.

### 3.3 Construction of a prognostic signature based on FAGs in osteosarcoma

**FIGURE 3:**
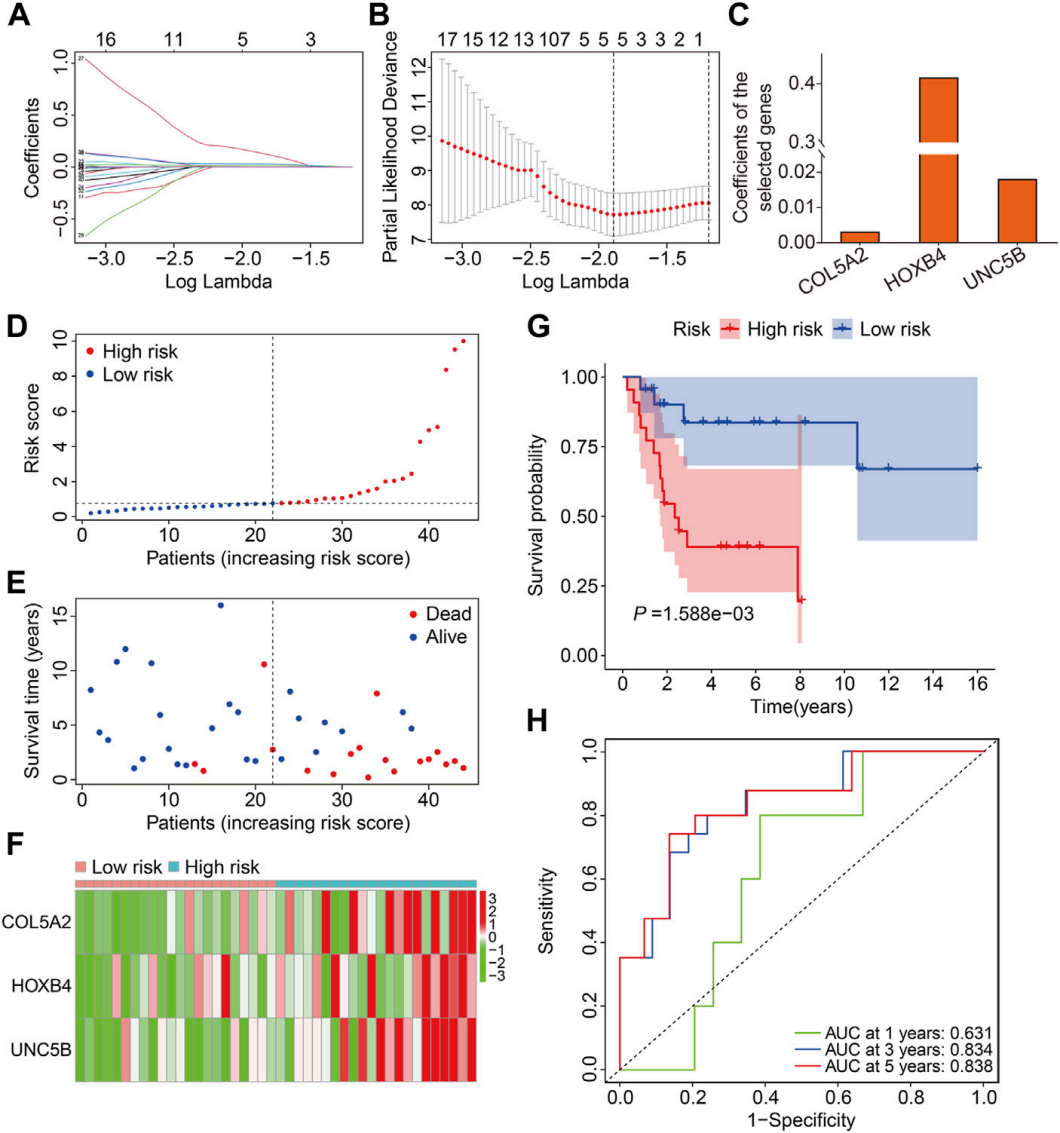
Construction of a prognostic signature in osteosarcoma based on ferroptosis-associated genes in the turquoise module. **(A,B)** Lasso regression and multivariable Cox regression analyses were performed. **(C)** Regression coefficients of the three FAGs. **(D–F)** Risk score distribution, vital status, survival time distribution, and expression profiles of the three FAGs in high- and low-risk groups. **(G)** Kaplan–Meier survival analysis comparing overall survival between the risk groups. **(H)** Time-dependent ROC curves for 2-, 3-, and 5-year survival prediction.

We chose 71 FAGs in the turquoise module to develop a predictive signature for osteosarcoma because the turquoise module was strongly linked with patient prognosis. First, we randomly assigned patients in TCGA cohort, also named the entire cohort, to one of two cohorts: training (n = 44) and testing (n = 42). The 71 FAGs were employed in the LASSO regression analysis to determine the most accurate predictive FAGs in the training cohort (Figure 3A). Following multivariate Cox regression analysis, three FAGs (*COL5A2*, *HOXB4*, and *UNC5B*) were selected by further optimization analysis (Figure 3B), and their corresponding regression coefficients were calculated (Figure 3C). The prognostic signature was established by the linear combination of the expression levels of the three FAGs and their coefficients, and the patients’ risk score was computed using the following formula: risk score = *COL5A2* × 0.0032 + *HOXB4* × 0.4108 + *UNC5B* × 0.0181. Subsequently, the patients in the training cohort were stratified into high- and low-risk groups, according to the median risk score (Figure 3D). The vital status and survival time distribution of patients in the training cohort are shown in Figure 3E, which suggests that most of the deceased patients were distributed in the high-risk group. The expression levels of the three FAGs in the high- and low-risk groups are shown in Figure 3F. The Kaplan–Meier survival analysis indicated a significant difference between the risk groups, with patients in the high-risk group having a shorter survival time than those in the low-risk group (Figure 3G). Time-dependent receiver operating characteristic (ROC) curve analysis confirmed the favorable predictive and prognostic accuracy of the signature, and the area under the curve (AUC) values were 0.631, 0.834, and 0.838 for 1-, 3-, and 5-year survival, respectively (Figure 3H).

### 3.4 Validation of three FAG-based signatures in internal cohorts

**FIGURE 4:**
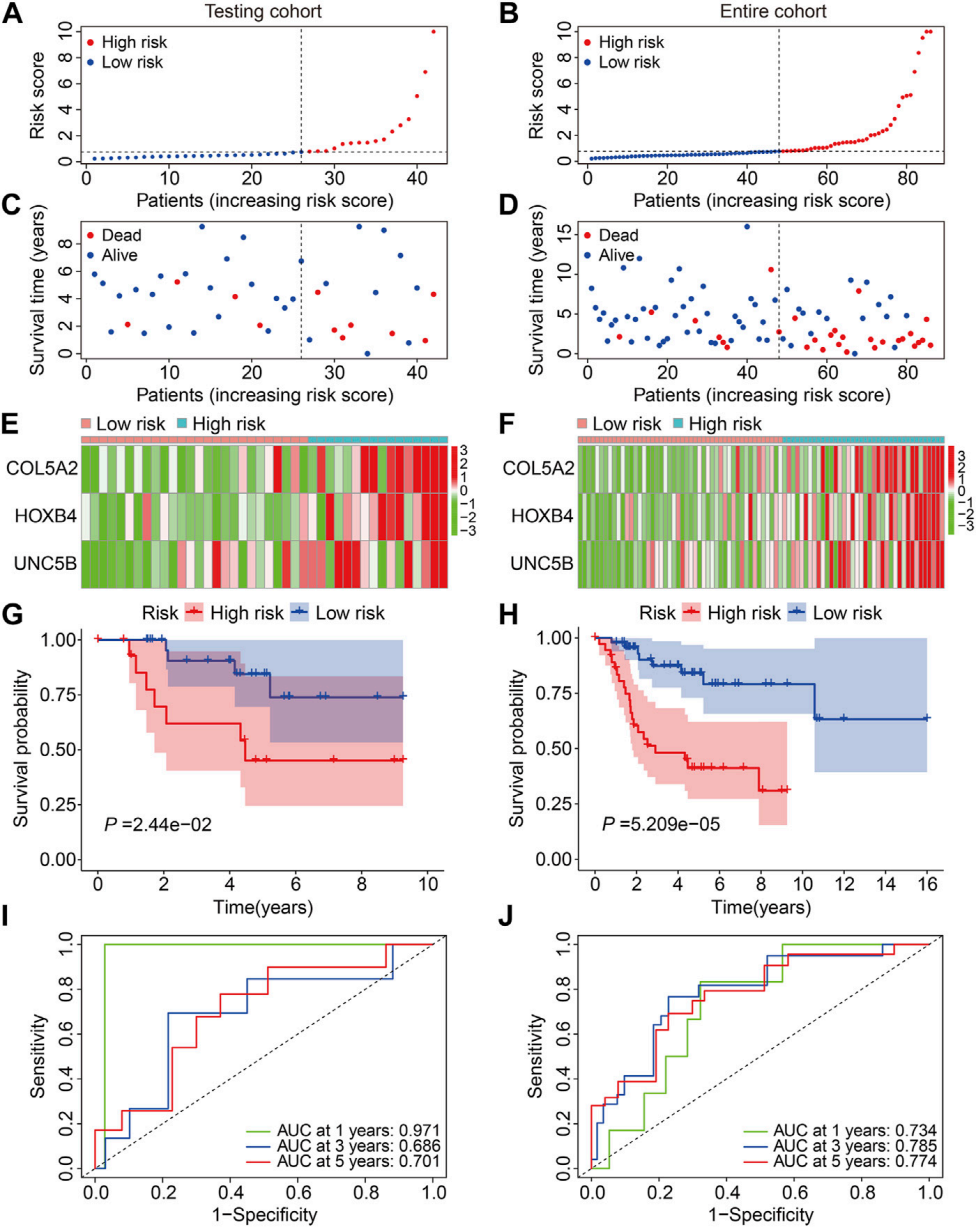
Validation of the three-gene-based prognostic signature in internal cohorts. **(A,B)** Risk score distribution in the testing and entire cohorts. **(C,D)** Vital status and survival time distribution in the testing and entire cohorts. **(E,F)** Expression profiles of the three FAGs in diverse risk groups of the testing and entire cohorts. **(G,H)** Kaplan–Meier survival analysis comparing overall survival between the risk groups in the testing and entire cohorts. **(I,J)** Time-dependent ROC curves for 2-, 3-, and 5-year survival prediction in the testing and entire cohorts.

First, we investigated the predictive capacity of the FAG-based signature by examining the testing and entire cohort datasets. Using the same formula mentioned previously, we computed the risk scores of patients in the testing and entire cohorts and then categorized them into high- and low-risk groups based on the median risk score value in the training cohort (Figures 4A,B). Figures 4C,D show the distribution of the vital status and survival time of patients in the testing and entire cohorts, and it seemed that patients in the high-risk group had a higher mortality rate in both cohorts. Figures 4E,F exhibit the expression profiles of the three FAGs in the different risk groups of the two cohorts. Kaplan–Meier survival analysis suggested that patients in the high-risk group had a worse prognosis than those in the low-risk group, which was consistent in both the testing and entire cohorts (Figures 4G,H). The AUC values of the ROC curves for 1-, 3-, and 5-year survival were 0.971, 0.686, and 0.701, respectively, in the testing cohort (Figure 4I) and 0.734, 0.785, and 0.774, respectively, in the entire cohort (Figure 4J).

### 3.5 Validation of three FAG-based signatures in the external cohort

**FIGURE 5:**
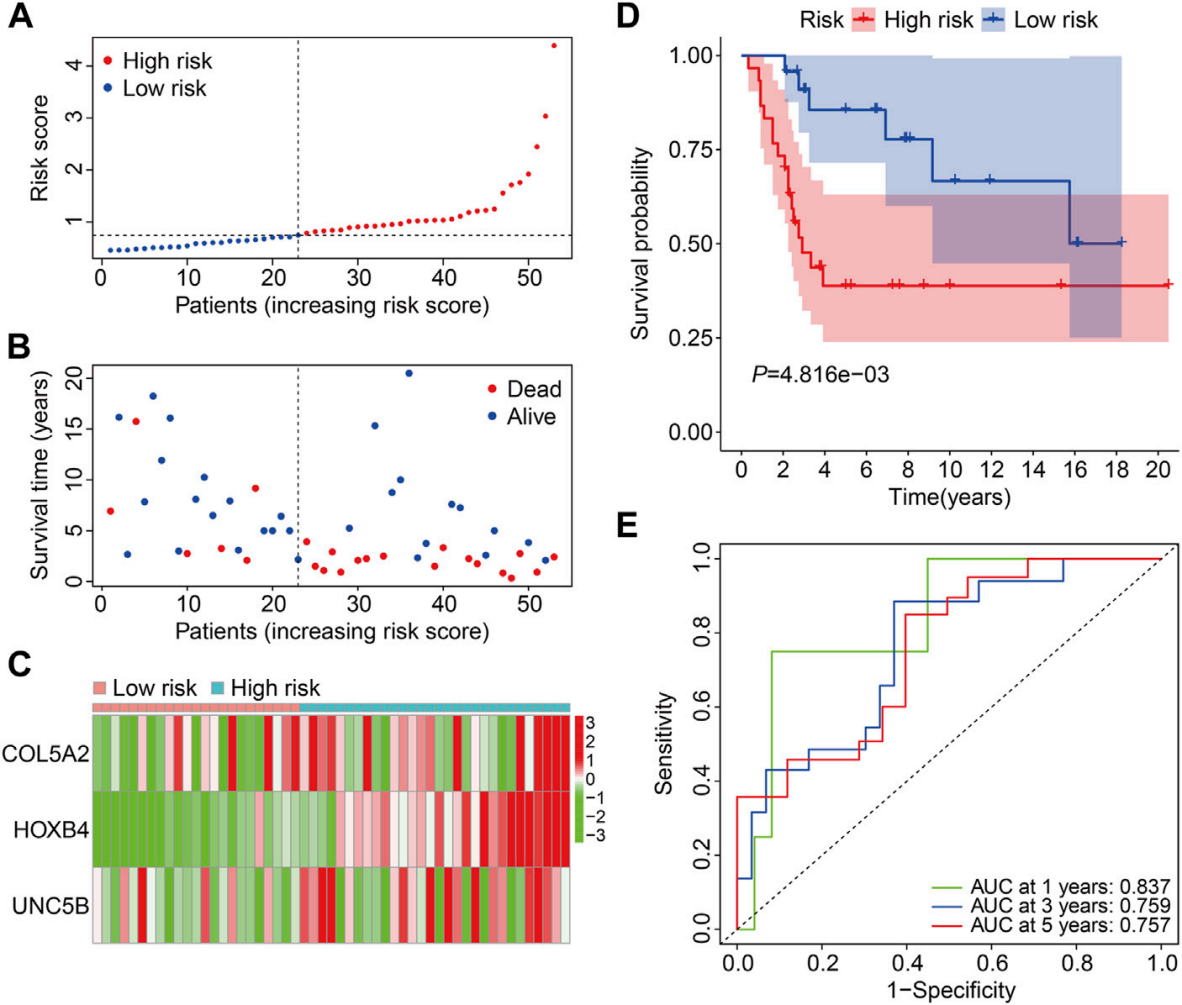
Validation of the three-gene-based prognostic signature in external GSE21257 cohorts. **(A)** Patients’ risk score distribution in the GSE21257 cohort. **(B)** Patients’ vital status and survival time distribution in the GSE21257 cohort. **(C)** Expression profiles of the three FAGs in diverse risk groups of the GSE21257 cohort. **(D)** Kaplan–Meier survival analysis comparing overall survival between risk groups in the GSE21257 cohort. **(E)** Time-dependent ROC curves for 2-, 3-, and 5-year survival prediction in the GSE21257 cohort.

**FIGURE 6:**
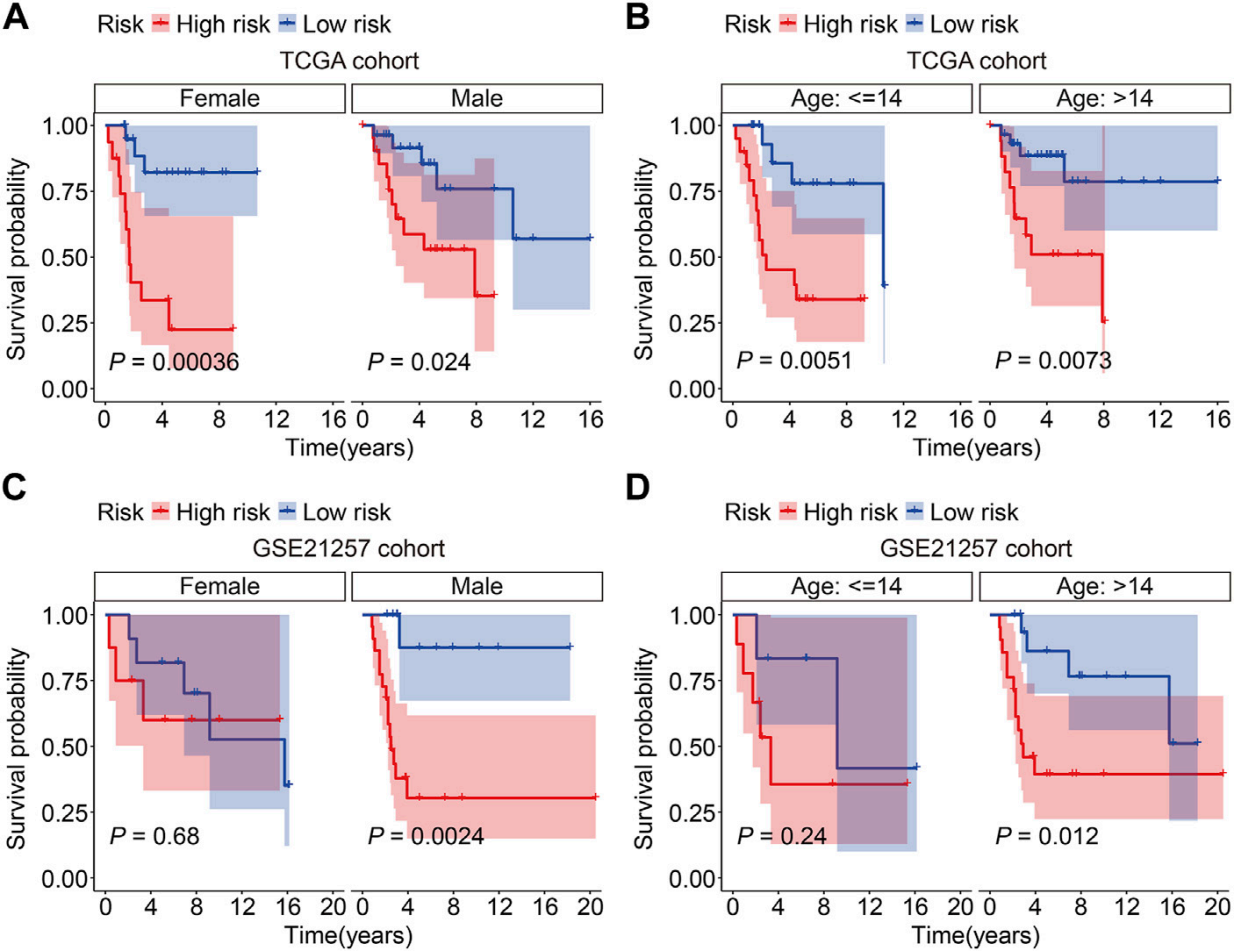
Kaplan–Meier survival curves in multiple subgroups stratified by gender **(A and C)** or age **(B and D)** in both TCGA and GSE21257 cohorts.

Subsequently, the GSE21257 cohort was used for external validation to further assess the accuracy and generalizability of the signature in predicting the prognosis of osteosarcoma patients. Patients’ risk scores were generated using the same formula as in the training cohort, allowing patients to be classified as high- or low-risk based on the median risk score value in the training cohort (Figure 5A). The vital status and survival time distributions of the GSE21257 cohort patients are shown in Figure 5B, and the majority of the deaths were in the high-risk categories. Figure 5C depicts the expression patterns of the three FAGs in the GSE21257 cohort’s distinct risk categories. The survival study revealed that patients with lower risk scores fared better in terms of OS than those with higher risk scores (Figure 5D). The AUC values of the ROC curves for the 1-, 3-, and 5-year survival in the GSE21257 cohort were 0.837, 0.759, and 0.757, respectively (Figure 5E). Furthermore, we divided the patients in the entire cohort and GSE21257 cohorts into various subgroups, including females and males aged ≤14 years and >14 years. As shown in Figures 6A,B, the survival analysis in different subgroups of the entire cohort revealed that the OS of patients in the high-risk group was continuously poorer than that of patients in the low-risk group. Additionally, the FAG-based signature performed well in predicting prognosis in subgroups such as male sex and age >14 years (Figures 6C,D). Taken together, these findings confirmed that the FAG-based signature has a potential prognostic value for patients with osteosarcoma.

### 3.6 The prognostic independence of the FAG-based signature and construction of a nomogram

**FIGURE 7:**
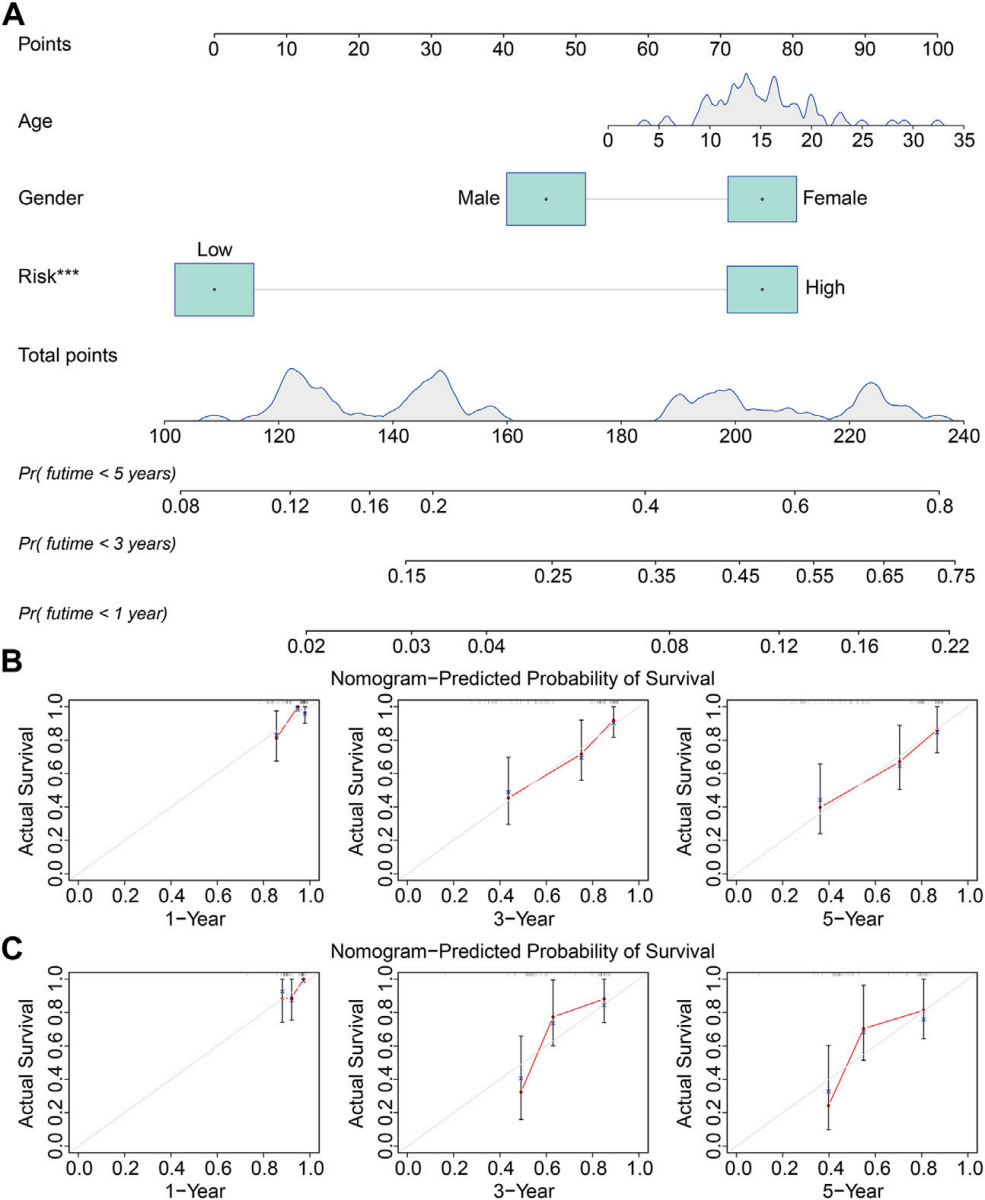
Construction of a prognostic nomogram for survival prediction. **(A)** Nomogram including gender, age, and signature-derived risk score for 1-, 3-, and 5-year overall survival rate prediction. **(B,C)** Calibration plot for predicting patients’ survival at 1, 3, and 5 years in TCGA cohort **(B)** and the GSE21257 cohort **(C)**.

We performed univariate and multivariate Cox regression analyses in TCGA and GSE21257 cohorts, using sex, age, and signature-derived risk score as explanatory factors to further examine the clinical value of the FAG-based signature in predicting the prognosis of osteosarcoma patients. In TCGA cohort, the risk score was the only factor that was significantly associated with the OS of osteosarcoma patients in both univariate and multivariate Cox regression analyses; a similar result was also seen in the GSE21257 cohort (Table 1 and Table 2). Therefore, we concluded that our signature-derived risk score had an independent effect on patient prognosis. Furthermore, we created a nomogram that combined the signature-derived risk score with several clinical variables such as sex and age to serve as a quantitative method for clinicians to anticipate the clinical outcomes of each case (Figure 7A). The calibration plots showed that the nomogram-predicted 1-, 3-, and 5-year OS rates were well-matched with the actual OS rates, and comparable results were seen in both TCGA and GSE21257 cohorts (Figures 7B,C). Thus, our nomogram has excellent predictive performance and clinical application value.

**TABLE 1:** Univariate and multivariate analyses of the three FAGs’ prognostic signature and clinical factors in TCGA cohort.

Variable	Univariate analysis				Multivariate analysis			
	HR	95% CI of HR		*P*	HR	95% CI of HR		*P*
		Lower	Upper			Lower	Upper	
Gender (female vs. male)	0.6810850	0.3276198	1.4158997	0.3036702	0.7501854	0.3513603	1.6017125	0.4576490
Age (≤14 vs. > 14)	0.6515076	0.3131828	1.3553175	0.2516082	0.7302900	0.3435631	1.5523307	0.4139532
Risk score	1.2324516	1.1251912	1.3499368	0.0000068	1.2315409	1.1241535	1.3491867	0.0000077

**TABLE 2:** Univariate and multivariate analyses of the three FAGs’ prognostic signature and clinical factors in the GSE21257 cohort.

Variable	Univariate analysis				Multivariate analysis			
	HR	95% CI of HR		*P*	HR	95% CI of HR		*P*
		Lower	Upper			Lower	Upper	
Gender (female vs. male)	1.4029846	0.5878958	3.3481546	0.4454741	1.2146461	0.4841790	3.0471482	0.6786014
Age (≤14 vs. > 14)	1.0091035	0.9752283	1.0441553	0.6029446	1.0153246	0.9809549	1.0508986	0.3867248
Risk score	1.7095053	1.1183881	2.6130537	0.0132570	1.7173220	1.0995653	2.6821461	0.0174441

### 3.7 Gene set enrichment analysis and immunological analysis

**FIGURE 8:**
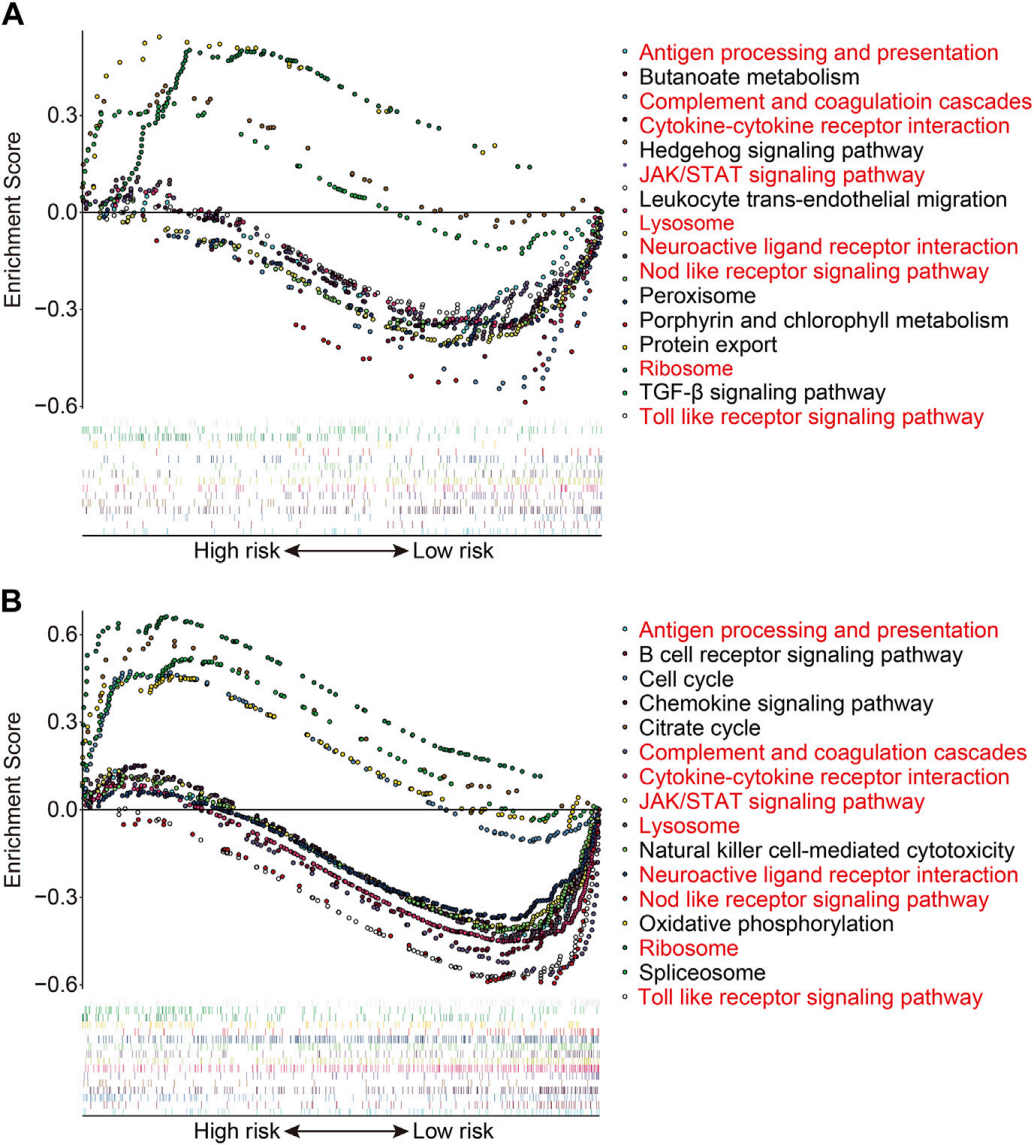
Gene set enrichment analysis between high- and low-risk groups in TCGA cohort **(A)** and the GSE21257 cohort **(B)**.

**FIGURE 9:**
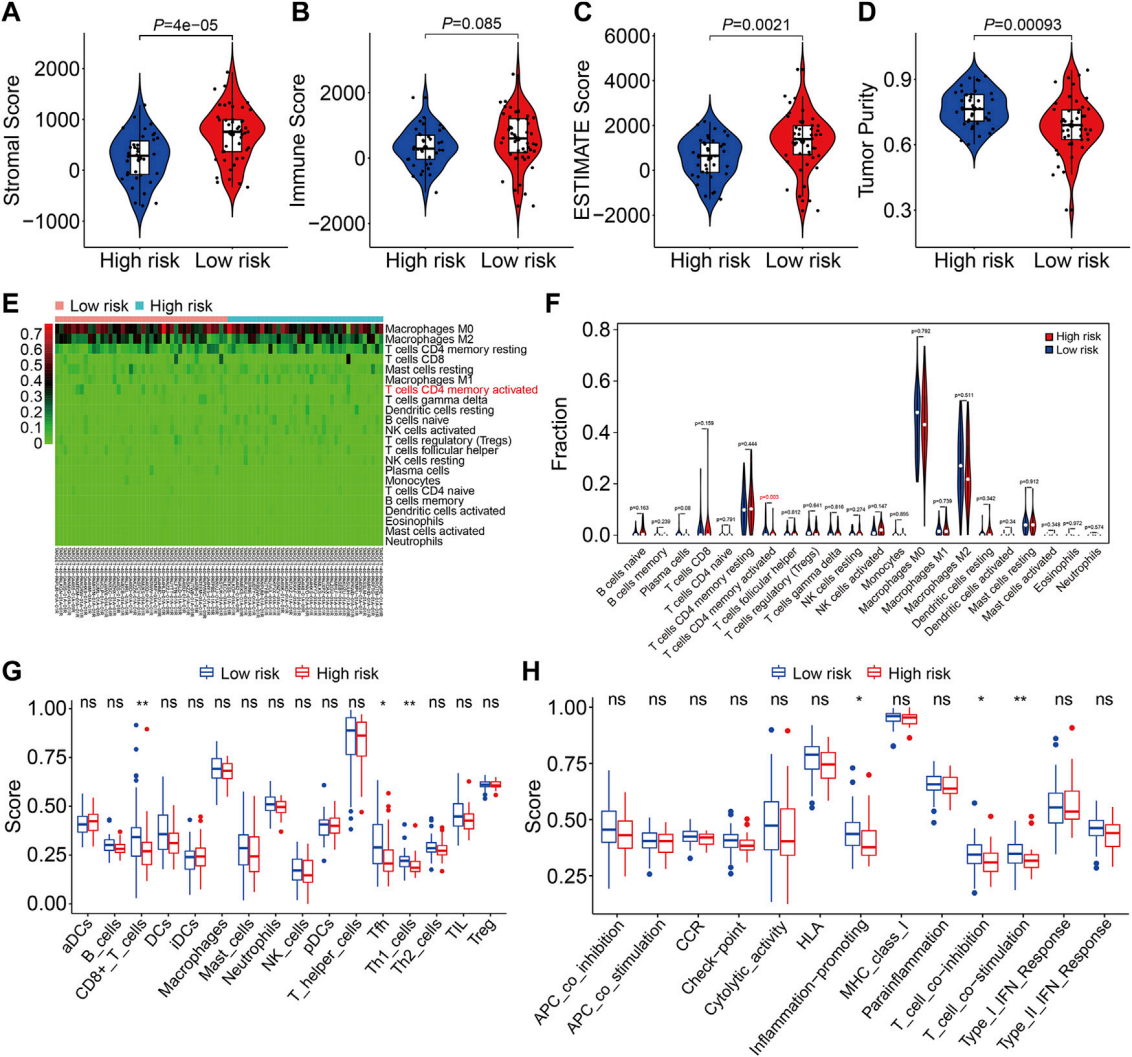
Estimation of the immune microenvironment and immune cell infiltration in high- and low-risk groups. **(A–D)** Comparison of the difference in stromal **(A)**, immune **(B)**, and ESTIMATE **(C)** scores, as well as tumor purity **(D)**, between high- and low-risk groups. **(E)** Heatmap showing the proportion of 22 immune cell types in TCGA osteosarcoma samples. **(F)** Comparison of the fraction of infiltrated immune cells between high- and low-risk groups. **(G,H)** Comparison of the scores of immune cells and immune-related function in high- and low-risk groups.

We used gene set enrichment analysis on datasets from different risk groups in TCGA and GSE21257 cohorts to identify pathways linked with the FAG-based signature. As shown in Figures 8A,B, ribosomes were significantly enriched in high-risk groups in both cohorts, whereas pathways including antigen processing and presentation, complement and coagulation cascades, the cytokine–cytokine receptor interaction, JAK/STAT signaling pathway, lysosomes, neuroactive ligand–receptor interaction, nod-like receptor signaling pathway, and toll-like receptor signaling pathway were significantly enriched in low-risk groups in both cohorts. Next, we explored the relationship of the signature with the immunological microenvironment and immune cell infiltration. The ESTIMATE method was used to compute stromal, immune, and ESTIMATE scores, as well as tumor purity. Significant variations in stromal and ESTIMATE scores, as well as tumor purity, were found when the high-risk and low-risk groups were compared (Figure 9A,C,D). Although the immune scores were lower in the high-risk group than in the low-risk group, the difference was not statistically significant (Figure 9B). Subsequently, we analyzed the proportion of infiltrated immunocytes in osteosarcoma samples from TCGA cohort. Figure 9E shows the profiles of 22 types of infiltrating immune cells in the high- and low-risk groups. Compared to the low-risk group, the proportion of activated CD4 memory T cells decreased significantly in the high-risk group (Figure 9F). Furthermore, we employed the ssGSEA method to calculate the scores for immune cells and immune-related functions. As shown in Figure 9G, the scores of CD8^+^ T, Tfh, and Th1 cells were different across the high- and low-risk groups and were consistently lower in the high-risk group than in the low-risk group. In terms of immune-related activities, the high-risk group had considerably lower scores for inflammation promotion, T-cell co-inhibition, and T-cell co-stimulation than the low-risk group (Figure 9H). When combined, these findings demonstrate the differential immunological state of the high- and low-risk groups.

### 3.8 Expression and Kaplan–Meier survival analyses of the three FAGs

**FIGURE 10:**
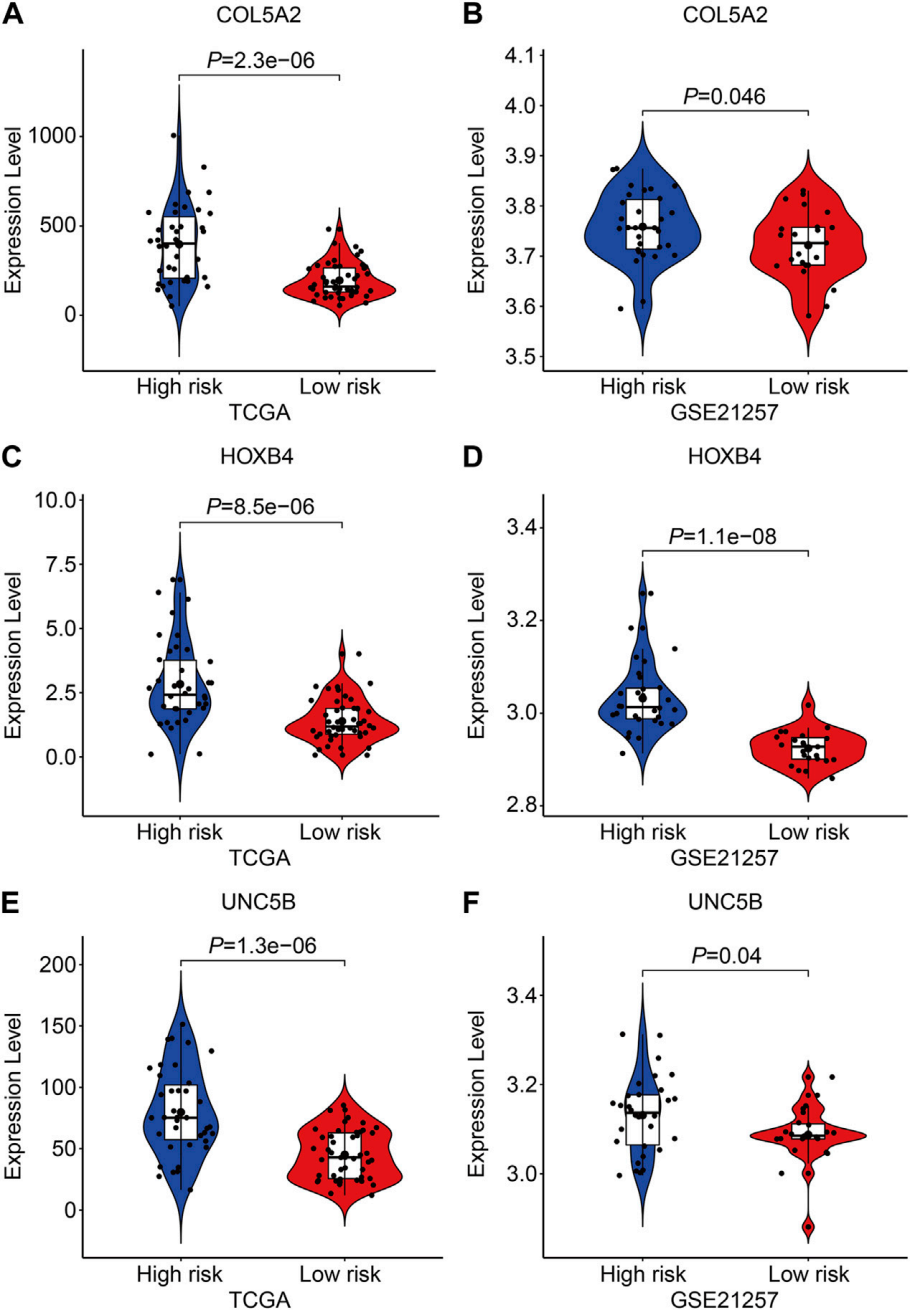
Comparison of the expression of *COL5A2* **(A,B)**, *HOXB4* **(C,D)**, and *UNC5B* **(E,F)** in different risk groups in TCGA and GSE21257 cohorts.

**FIGURE 11:**
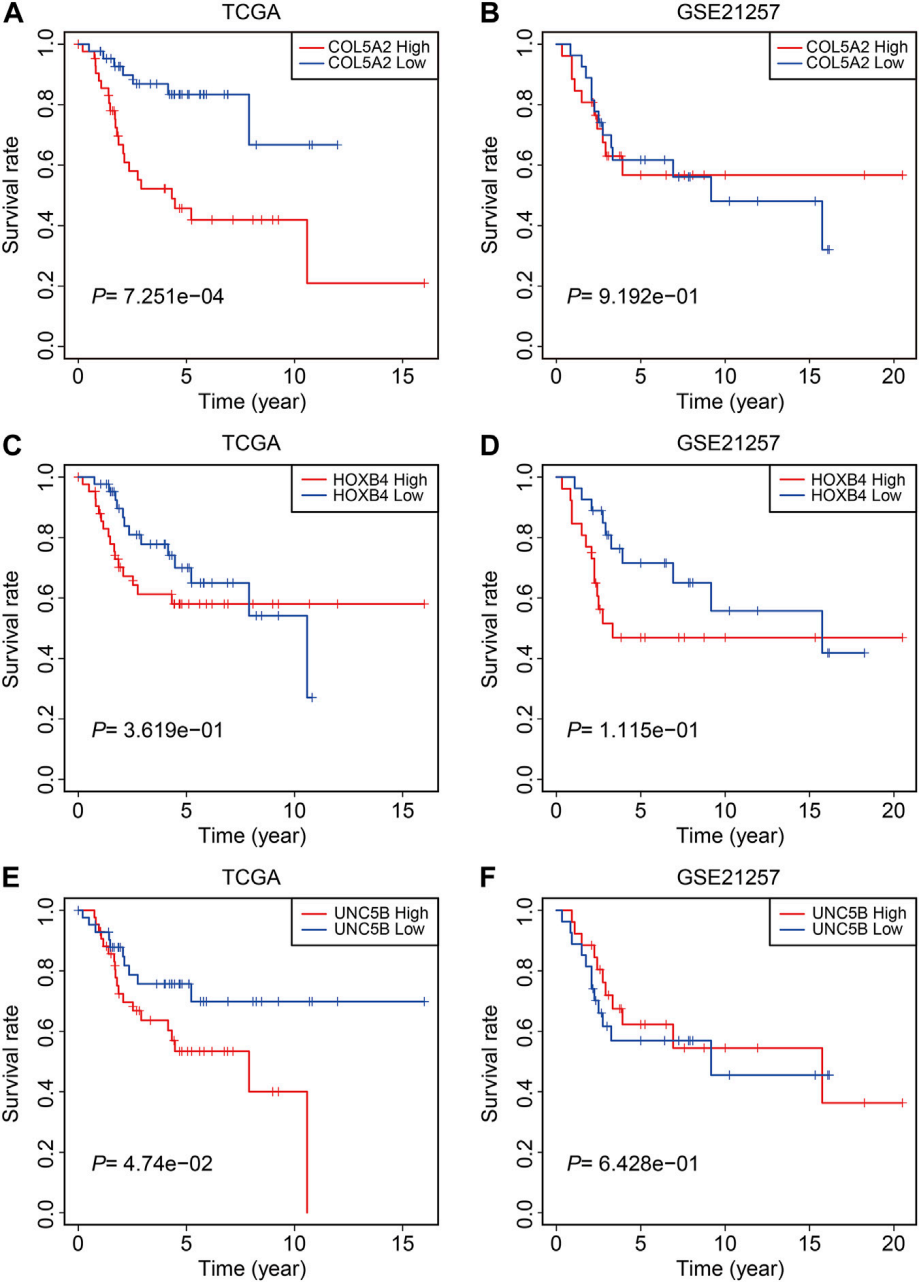
Kaplan–Meier survival analysis of *COL5A2* **(A,B)**, *HOXB4* **(C,D)**, and *UNC5B* **(E,F)** in TCGA and GSE21257 cohorts.

**FIGURE 12:**
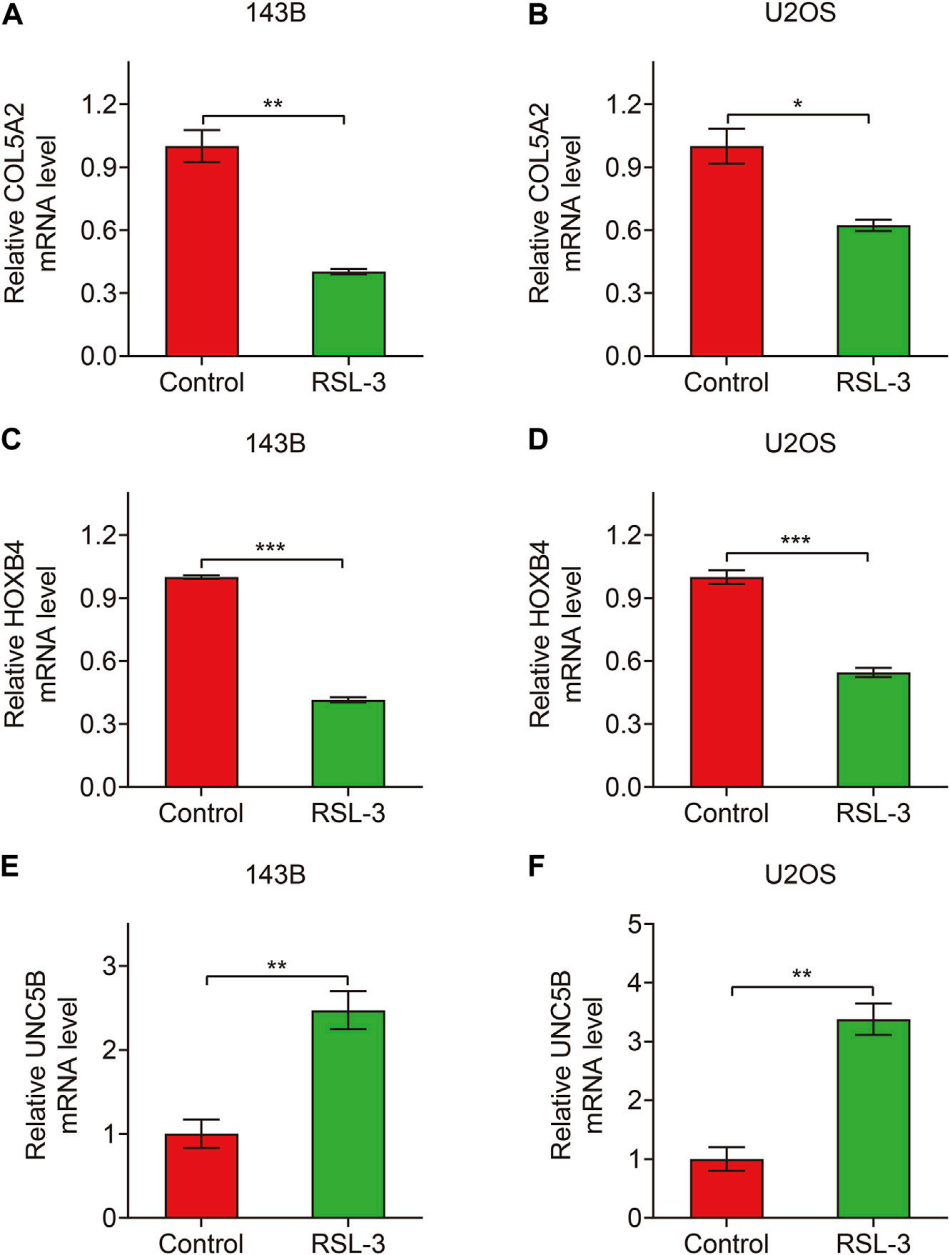
Relative expression of *COL5A2* **(A,B)**, *HOXB4* **(C,D)**, and *UNC5B* **(E,F)** in control and RSL3-treated osteosarcoma cells.

Finally, in TCGA and GSE21257 cohorts, we analyzed the expression of the three FAGs in the high- and low-risk groups and performed Kaplan–Meier survival analysis. The expression of *COL5A2*, *HOXB4*, and *UNC5B* was greater in the high-risk group than that in the low-risk group, as shown in Figures 10A–F, and this was consistent in both TCGA and GSE21257 cohorts. In TCGA cohort, Kaplan–Meier survival analysis revealed that increased *COL5A2* and *UNC5B* expression indicated a worse prognosis in patients with osteosarcoma (Figures 11A,E), but *HOXB4* expression was not linked with patients’ clinical fate (Figure 11C). In the GSE21257 cohort, the survival analysis revealed no significant association between the three FAGs and patient outcomes (Figures 11B,D,F). We also compared the expression levels of the three FAGs in RSL3-treated osteosarcoma cells and control cells. Consistent with the RNA-seq data, the mRNA levels of *COL5A2* and *HOXB4* were lower and *UNC5B* mRNA levels were higher in RSL3-treated cells than in control cells (Figures 12A–F), indicating the probable roles of the three FAGs in the ferroptotic processes of osteosarcoma cells.

## 4 Discussion

Recent research has shown that ferroptosis plays an important role in neurodegenerative disorders, ischemic stroke, traumatic brain injury, acute kidney injury, and tumors (DeGregorio-Rocasolano et al., 2019; Li et al., 2019; Mou et al., 2019; Reichert et al., 2020; Wang et al., 2021b). Owing to its novelty and importance, ferroptosis is now the focus of research to improve the treatment outcomes of certain diseases, especially tumors. Numerous studies have confirmed that inducing ferroptosis can effectively inhibit malignant tumors, and many genes associated with ferroptosis might serve as potential targets for clinical applications. For example, Badgley et al. (2020) found that cystine is critical for pancreatic ductal adenocarcinoma (PDAC). Genetic knockout of Slc7a11, a subunit of the cysteine–-glutamate antiporter, induces intracellular cysteine depletion, thereby inducing ferroptosis and inhibiting PDAC. Therefore, Slc7a11 is a potential target for PDAC treatment. In osteosarcoma, the induction of ferroptosis with traditional Chinese medicine and small molecules could enhance sensitivity to chemotherapy (Liu and Wang, 2019; Lin et al., 2021; Luo et al., 2021), thus providing a new avenue for the treatment of chemo-resistant patients. However, little is known about the regulatory mechanism of ferroptosis in osteosarcoma and its clinical significance.

Here, we systematically analyzed the transcriptional changes in osteosarcoma cells induced by RSL3 and found that 728 genes were differentially expressed between the control and RSL3-treated cells. Of the 728 genes, 450 and 278 genes were upregulated or downregulated in RSL3-treated cells, respectively, and were regarded as ferroptosis-associated genes (FAGs) in osteosarcoma. Functional enrichment analysis indicated that FAGs were mainly enriched in PI3K-Akt, MAPK, calcium, JAK-STAT, TNF, IL-17, Hippo, and NF-κB signaling pathways and ferroptosis pathways. It is worth noting that these enriched pathways have been reported to promote or inhibit the ferroptotic process in multiple tumor types (Chen et al., 2020; Yi et al., 2020; Schmitt et al., 2021; Yu et al., 2022); however, their roles in the ferroptosis of osteosarcoma and the regulatory mechanism remain to be further elucidated.

Subsequently, WGCNA was performed using the mRNA expression of FAGs and clinical data on osteosarcoma samples in TCGA dataset, and we obtained a turquoise module that was markedly associated with the vital status of osteosarcoma patients. Subsequently, 71 FAGs in the turquoise module were subjected to LASSO and multivariate Cox regression analysis for further optimization and screening. A total of three FAGs, *COL5A2*, *HOXB4*, and *UNC5B*, were ultimately used to construct a prognostic signature for osteosarcoma. The risk score of each patient was then calculated based on the signature, which allowed the patients to be stratified into high- and low-risk groups. The survival analysis in all internal and external cohorts demonstrated that the OS of patients in the high-risk group was continuously poorer than that of patients in the low-risk group, validating the accuracy and clinical applicability of the signature in predicting patient prognosis. Univariate and multivariate Cox regression analyses suggested that the signature-derived risk score is an independent indicator of patient prognosis. Moreover, we built a prognostic nomogram by combining risk scores and clinical variables, such as sex and age. The nomogram could serve as a quantitative method for clinicians to anticipate the clinical outcomes of each case and might be helpful for clinical decision-making.

The tumor microenvironment (TME) is a component of cancer that includes the extracellular matrix, stromal cells, fibroblasts, endothelial cells, and several types of immune cells (Cassim and Pouyssegur, 2019; Hinshaw and Shevde, 2019). Recent research has concluded that tumor growth is influenced not only by the accumulation of genetic or epigenetic abnormalities in the initial cancer cells but also by the TME (Duan et al., 2020). Immune cells in the TME were found to be associated with patient prognosis and possibly operate as a prognostic factor and influence immunotherapy response (Hu et al., 2020; Lei et al., 2020). To characterize the immune landscape of osteosarcoma, we used the ESTIMATE method to evaluate stromal and immune microenvironment infiltration in osteosarcoma samples from the different risk groups. Significant variations in stromal and ESTIMATE scores, as well as tumor purity, were found between the high- and low-risk groups. As for infiltrating immune cells, the fraction of activated CD4 memory T cells was much lower in the high-risk group. Activated CD4 memory T cells are transformed from CD4 memory T cells after antigen stimulation, resulting in a more effective and rapid immune response. The abundance of activated CD4 memory T cells has been reported to be associated with patient prognosis in multiple solid tumors, such as colorectal cancer, small-cell lung cancer, and hepatocellular carcinoma (Jiang et al., 2021; Liu et al., 2021; Yu and Zhu, 2021). Consistently, our analysis connected a lower proportion of infiltrated activated CD4 memory T cells with a worse prognosis in osteosarcoma. Additionally, the ssGSEA results suggested that the enrichment scores of CD8^+^ T, Tfh, and Th1 cells were lower in the high-risk group than those in the low-risk group. In terms of immune-related activities, the high-risk group had considerably lower scores for inflammation promotion, T-cell co-inhibition, and T-cell co-stimulation than the low-risk group. Taken together, these results demonstrate the differential immunological state of the high- and low-risk groups. Our FAG-based signature may be useful for distinguishing patients into different immunological subgroups and predicting the clinical effects of immunotherapy.

Our signature comprised three genes, *COL5A2*, *HOXB4*, and *UNC5B*, which were confirmed to be altered in transcript levels, following treatment with the ferroptosis inducer, RSL3. *COL5A2,* also known as collagen type V alpha 2, is located at 2q32.2 in the human genome and encodes an alpha chain of fibrillar collagen type V. *COL5A2* functions as an oncogene in multiple tumor types, and higher *COL5A2* expression indicates poor prognosis in patients with colorectal cancer (Wang et al., 2021a), gastric cancer (Ding et al., 2021), and bladder cancer (Zeng et al., 2018). *COL5A2* was reported to be highly expressed in metastatic osteosarcoma, and silencing *COL5A2* impaired cell invasion and metastasis by inhibiting the TGF-β and Wnt/β-catenin signaling pathways (Han et al., 2022). Here, our analyses showed that *COL5A2* expression was higher in the high-risk group, and increased *COL5A2* expression indicated a worse prognosis in osteosarcoma patients, suggesting a potential oncogenic role in osteosarcoma. Additionally, we found that *COL5A2* was decreased in RSL3-treated cells. Thus, we speculated that *COL5A2* might participate in the regulation of the ferroptotic process in osteosarcoma. Further experiments should be designed to explore the effects of *COL5A2* knockdown or overexpression on RLS3-induced ferroptosis in osteosarcoma cells. *HOXB4*, a member of the homeobox gene family, is a transcription factor involved in stem cell self-renewal and cancer (Shah and Sukumar, 2010; Feng et al., 2021). In several forms of solid tumors, *HOXB4* acts as both an oncogene and a tumor suppressor. For example, in ovarian cancer, increased *HOXB4* expression was associated with a worse prognosis, and *HOXB4* overexpression enhanced the malignant evolution of ovarian cancer through the transcriptional regulation of the dehydrodolichyl diphosphate synthase subunit (DHDDS) (Li et al., 2020b), whereas increased *HOXB4* expression in cervical cancer obviously inhibited cell proliferation and reduced tumorigenic potential (Lei et al., 2021). The HOXB4 protein was found in more than 90% of neoplastically transformed cells in osteosarcoma (Bodey et al., 2000); however, its significance is still unknown and requires further research. *UNC5B*, also known as UNC-5 homolog B, is a member of the UNC5 receptor family. *UNC5B* is located on chromosome 10q22.1, which encodes a single-pass transmembrane receptor protein that is noted for its unusual capacity to produce two opposing intracellular signals in the presence or absence of ligands (Bhat et al., 2019). *UNC5B* has the potential to either promote or prevent tumor growth in certain tumor types (Okazaki et al., 2012; Wu et al., 2020; Huang et al., 2021). In osteosarcoma U2OS cells, doxycycline-induced expression of polyomavirus small T antigen (PyST) resulted in mitotic arrest and extensive cell death, accomplished by increased mRNA levels of UNC5B, showing the apoptotic activity of UNC5B in osteosarcoma (Bhat et al., 2020). Our findings revealed that UNC5B expression was associated with patient prognosis and that UNC5B mRNA levels were elevated in ferroptotic osteosarcoma cells. Given UNC5B’s dual involvement in malignancies, it is worth investigating its role in osteosarcoma, particularly its influence on the ferroptotic process.

Despite the aforementioned findings, our study had some limitations. First, some important regulators of ferroptosis function at the protein level, whereas their mRNA expression might be stable during RSL3-induced ferroptosis, and these genes were excluded from further analysis in the present study. Second, prospective cohorts are needed to assess the clinical utility of our signature. In addition, we only detected the mRNA levels of the three FAGs in RSL3-treated cells; it would be better to explore their protein levels after the treatment of RSL3, and the role of these three FAGs in the ferroptotic process of osteosarcoma cells should be investigated *in vitro* and *in vivo*.

In summary, we systematically analyzed transcriptional changes in osteosarcoma cells induced by RSL3 and constructed a novel three-gene signature with regard to ferroptosis, prognosis prediction, and the immune microenvironment. We identified three FAGs (*COL5A2*, *HOXB4*, and *UNC5B*) as potential therapeutic targets and important regulators of ferroptosis in osteosarcoma.

## Data Availability

The original contributions presented in the study are included in the article/Supplementary Materials; further inquiries can be directed to the corresponding author.
