# Supplementary material for: Identification of a novel gene signature with regard to ferroptosis, prognosis prediction, and immune microenvironment in osteosarcoma
Source: Front Genet. 2022 Oct 18;13:944978. doi: 10.3389/fgene.2022.944978 (PMC9623102; doi:10.3389/fgene.2022.944978)
Supplement: Supplementary file 1 [file Table1.DOCX]

Supplementary Material


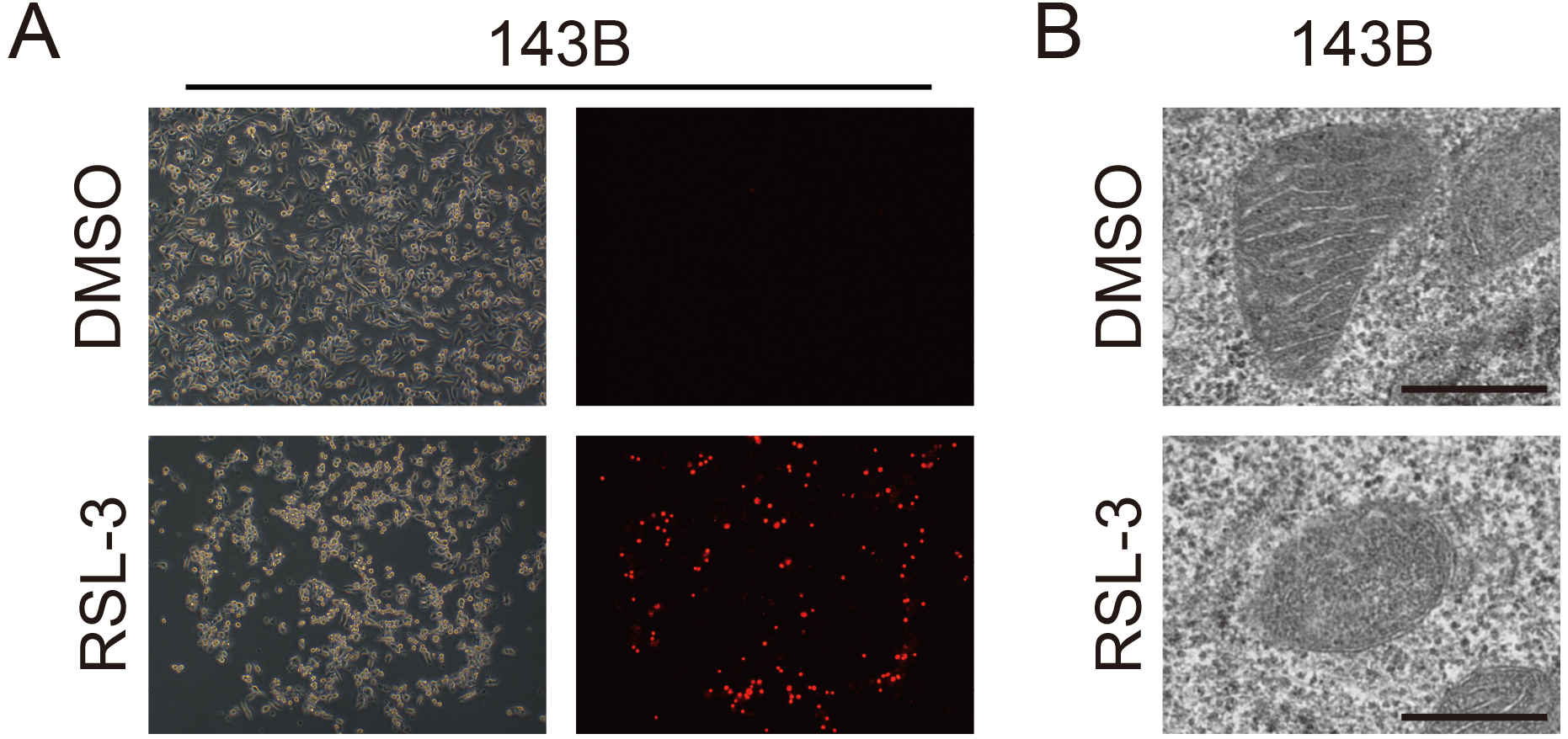


**Supplementary Figure 1**. RSL3 induced ferroptosis in osteosarcoma cells. (A) PI staining of 143B cells treated with RSL3. (×100). (B) Transmission electron microscopy was performed to evaluate the morphological feature of mitochondria of 143B cells under RSL3 treatment after 24 h. Scale bar, 500 nm.
